# Population-Based 3D Mapping of Inferior Alveolar Nerve Clearance in Bilateral Sagittal Split Osteotomy

**DOI:** 10.3390/jpm16050237

**Published:** 2026-04-30

**Authors:** Haye H. Glas, Tom L. Zwijnenberg, Johan Jansma, Rutger H. Schepers

**Affiliations:** 1 Clinic for Virtual Surgical Planning, 3D VSP B.V., Hoogwatum 2, 9906 TD Bierum, Groningen, The Netherlands; 2 Department of Oral and Maxillofacial Surgery, Expertcenter for Orthofacial Surgery, Martini Hospital Groningen, Van Swietenplein 1, 9728 NT Groningen, Groningen, The Netherlands

**Keywords:** bilateral sagittal split osteotomy, mandibular canal, cortical bone thickness, inferior alveolar nerve, statistical shape model

## Abstract

**Background/Objectives**: Inferior alveolar nerve injury is a common complication of Bilateral Sagittal Split Osteotomy. Preoperative three-dimensional tracing of the mandibular nerve canal using Cone-beam CT may help reduce this risk. We reconstructed the nerve course in 428 consecutively planned BSSO cases and developed a statistical model that quantifies population-level canal position to guide safe, evidence-based osteotomy planning. **Methods**: Traceable mandibular nerve canal CBCTs from 440 BSSO candidates (2023–2025) were retained. The mandibles and 2.5 mm diameter canals were segmented in Mimics and fused with intraoral scans. Next, the meshes were aligned non-rigidly to a template. A k-nearest-neighbour analysis mapped the outer mandibular surface to canal distances of all the patients on the template mandible model. **Results**: After excluding 12 scans because the nerve could not be traced, 428 mandibles were studied. The canal’s position varied, with regions near the vertical ramus and premolar regions frequently showing fewer than 3 mm of buccal bone covering the mandibular nerve. In contrast, a preferred safe zone was identified in the second molar region, where more than 97.8% of patients had greater than 5 mm of buccal bone clearance. A population-based colour map was generated to visualise the risk areas and confidence intervals for outer cortex-to-canal distances. **Conclusions**: This study provides the first high-resolution, population-based 3D map of nerve clearance in BSSO patients. Routine use of CBCT with patient-specific nerve tracing is recommended to reduce the risk of nerve injury.

## 1. Introduction

Bilateral Sagittal Split Osteotomy (BSSO) is a widely used procedure for correcting mandibular deformities. By separating the tooth-bearing distal segment from the proximal rami, the surgeon can advance, set back, or vertically reposition the mandible to restore occlusion and facial balance. During a BSSO corticotomy, the surgeon works in close proximity to the inferior alveolar nerve (IAN), which enters the mandible at the lingual mandibular foramen, courses anteriorly through the mandibular nerve canal, and exits at the mental foramen.

Injury to the IAN is a relatively common complication of BSSOs. Verweij et al. found a mean pooled incidence of 33.9% neurosensory disturbance after a BSSO [1]. Such disturbance may arise indirectly from traction or manipulation during splitting and fixation, or directly from mechanical trauma to the nerve [2]. Distinguishing the relative prevalence of these two mechanisms is challenging because most clinical series only document assessment of altered nerve sensation [3]. Complete transection of the IAN has been reported to occur in roughly 1.5% of sagittal split procedures [4]. Given these risks, detailed preoperative planning is essential to identify the mandibular canal’s course and to avoid direct nerve damage during a BSSO [5].

The mandibular nerve canal’s buccolingual position varies along the mandible. In a cadaveric study of 101 mandibles, Promma et al. measured the bone thickness between the canal and the outer cortices at several cut sites (ramus, third, second, and first molar, and premolar regions). They found that the available cortical bone for a safe split differs per location. At the mandibular ramus, the canal lies very close (2–3 mm) to the buccal cortex, whereas in the molar region, there is typically more buccal bone (5–6 mm) covering the nerve [6]. Similar findings have been reported in Cone-Beam Computed Tomography (CBCT) studies by measuring the distance to the outer mandibular surface at several locations [7,8]. The superior-inferior position of the nerve is equally variable. Promma et al. found in their cadaveric study that the canal dips to its lowest point near the first molar, with the canal sometimes only running 5 mm above the inferior border in that region [6]. The trajectory of the IAN is three-dimensionally complex and follows, what has been called, ‘a wavy pattern’ in a posterior to anterior direction [9].

Previous CBCT-based studies have typically measured canal-to-cortex distances at discrete cross-sectional slices or predefined anatomical landmarks. While valuable, this approach does not capture the continuous spatial variability of the nerve’s position across the mandible. A comprehensive three-dimensional analysis that statistically aggregates data across a large patient population to generate continuous risk maps has, to our knowledge, not yet been reported. The anatomical variability of the mandibular nerve canals’ course directly impacts BSSO planning. When the IAN runs very buccally or near the inferior border, it might be endangered by a corticotomy. Routine CBCT–based virtual surgical planning now allows surgeons to trace the canal of the IAN three-dimensionally and measure its clearance from the buccal and inferior cortices along the osteotomy line. Over the last few years, we have incorporated this nerve-mapping step into every BSSO plan, creating a consecutive dataset that captures anatomic variability. The current study was undertaken to analyse a large real-world dataset of consecutively planned CBCT-based BSSO cases to define safe corridors for an osteotomy and to test assumptions about minimum canal-to-cortex clearance requirements. By statistically evaluating the IAN’s position on preoperative CBCT scans, we aimed to quantify how much bone covers the mandibular nerve canal at key BSSO cut sites, and to identify scenarios where the available bone thickness may be insufficient. The aim of this study was to map population-based anatomical risk zones along the mandible and to identify regions where a buccal osteotomy can be performed with a wider safety margin. While patient-specific planning remains essential, these generalisable data may help guide osteotomy design.

## 2. Materials and Methods

### 2.1. Study Design and Patient Selection

The CBCT scans of patients for whom a BSSO was planned in the years 2023–2025 were considered for inclusion. CBCT scans were excluded if the nerve could not be reliably traced, for example, due to motion artifacts or other image quality problems.

### 2.2. Generation of 3D Models

The 3D models of all the patients’ mandibles were augmented with the intraoral scan of the dentition to obtain 3D models of the left and right mandibular nerve canals. The 3D models were made beforehand by an experienced technical physician (HG) using Materialise Mimics (Mimics Medical 26.0; Materialise NV, 99 Leuven, Belgium). The mandibular nerve canal was assumed to have a uniform diameter of 2.5 mm over its entire trajectory, aligning with a recent MRI-based morphometric study reporting median mandibular nerve diameters of approximately 2.0–2.5 mm in healthy individuals [10]. All 3D models were verified by the operating surgeon and subsequently anonymised.

### 2.3. Placing Corresponding Landmarks

To obtain matching anatomical landmarks from all the mandibles, the 3D models of the mandibles were non-rigidly aligned in Materialise 3-Matic (3-Matic Medical 18.0; Materialise NV, 99 Leuven, Belgium). This resulted in a set of mandibles with point-to-point correspondence, meaning all mandibles shared matching anatomical landmarks. To verify the result, the mandible models were visually checked to make sure there was no mesh distortion. A mean mandible shape was reconstructed for visualisation purposes.

### 2.4. Analysis

Every patient’s mandible and corresponding canals were represented as 3D surface meshes. For each mandible, the shortest distance from every cortical surface vertex to the outer surface of the mandibular nerve canal was calculated using VTK’s implicit-distance functionality in Python (version 3.14.4), resulting in a per-vertex list of distances for each individual.

All the mandibles had corresponding surface meshes with identical point ordering and vertex counts, which enabled pointwise aggregation. Mean distances at all surface points were computed across all patients. We also computed the 95% confidence interval of the mean at each surface point to assess the statistical precision of the estimated average distances. These values were visualised on a mean mandible model using colour maps (VTK’s toolkit Python).

To evaluate whether parametric summaries were appropriate, we tested the vertex-wise distance distributions for normality using the Shapiro–Wilk test (*p* = 0.05). The *p*-values were adjusted with the Benjamini–Hochberg false-discovery-rate (FDR) correction (α = 0.05) function. After correction, a substantial proportion (70%) of surface points still showed significant deviation from normality. Therefore, we derived a fully non-parametric boundary for defining surgical safety margins. The 2nd percentile of the distance distribution at each vertex was used, meaning at least 98% of patients had a larger nerve-to-cortex clearance at that location. The 2nd percentile of the distance distribution was visualised as a colour-coded plot on the mean mandible shape, identifying the regions with consistent clearance, and hence the regions where osteotomies can be safely performed in most patients.

### 2.5. Osteotomy-Specific Distance Analysis

**Figure 1:**
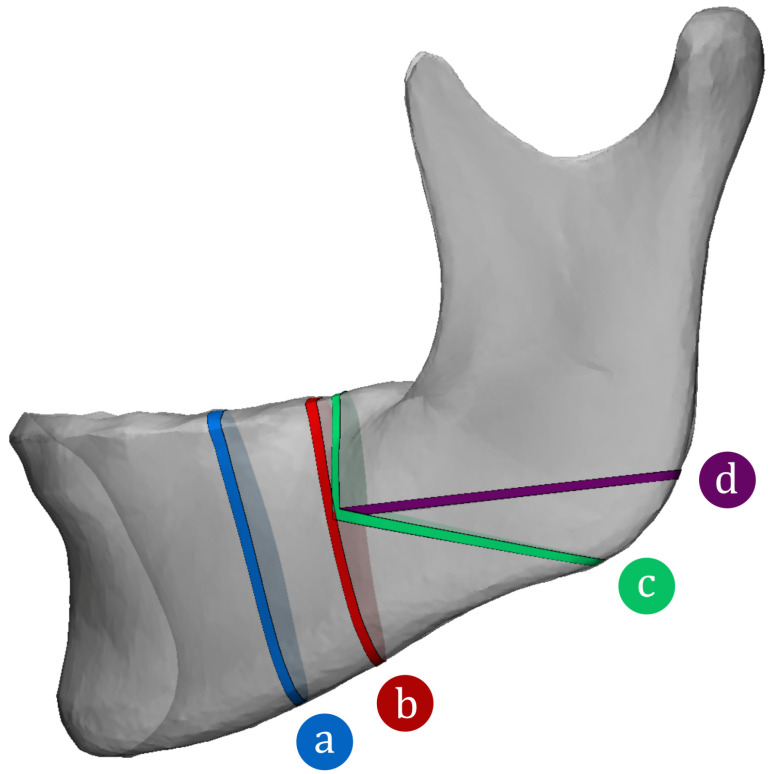
Four BSSO osteotomy trajectories projected onto the mean mandible model: (a) anterior, (b) pre-notch, (c) post-notch, and (d) post-gonial. Buccal and inferior clearance measurements to the mandibular nerve canal were obtained along each trajectory.

To provide clinically relevant measurements along commonly used BSSO osteotomy designs, we defined four osteotomy trajectories with the following classifications: premolar, pre-notch, post-notch, and post-gonial [5] (Figure 1). Along each osteotomy trajectory, the shortest distance from the cortical surface to the mandibular nerve canal was measured in two directions: buccally (perpendicular to the buccal cortex) and inferiorly (perpendicular to the inferior border). These measurements were performed bilaterally for each patient. The mean, 95% confidence interval of the mean, and 2nd percentile values were calculated for each osteotomy design.

## 3. Results

### 3.1. Study Data

**Figure 2:**
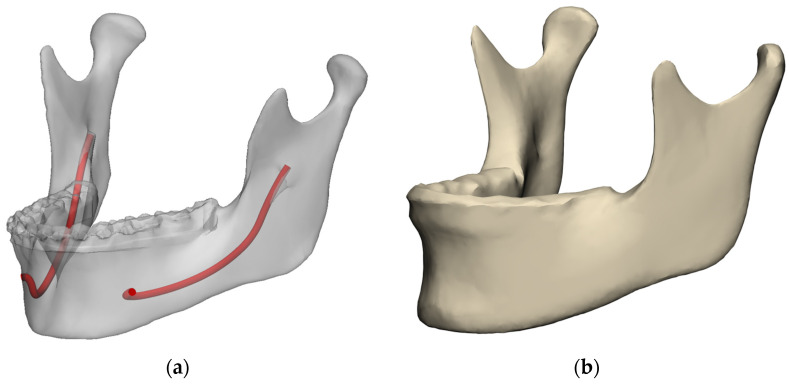
(**a**) Digital model of a patient’s mandible augmented with the intraoral dental scan and segmented mandibular nerve canals. (**b**) The mean mandible shape of 428 mandibles.

In total, the CBCT scans of 440 patients made between February 2023 and March 2025 were considered for inclusion. The corresponding STL models of the augmented mandible and bilateral mandibular nerve canals were retrieved for each scan. Twelve scans were excluded because the nerve was untraceable on one side. The faces and vertices of the remaining 428 digital mandible models were brought into correspondence, and a mean model was calculated using 3-Matic (Figure 2a,b). Figure 2a shows the mandible and traced nerves of one patient. Figure 2b shows the resulting mean mandible shape from the 428 patients.

### 3.2. Mapping of Inferior Alveolar Nerve Canal Distance to Cortex

**Figure 3:**
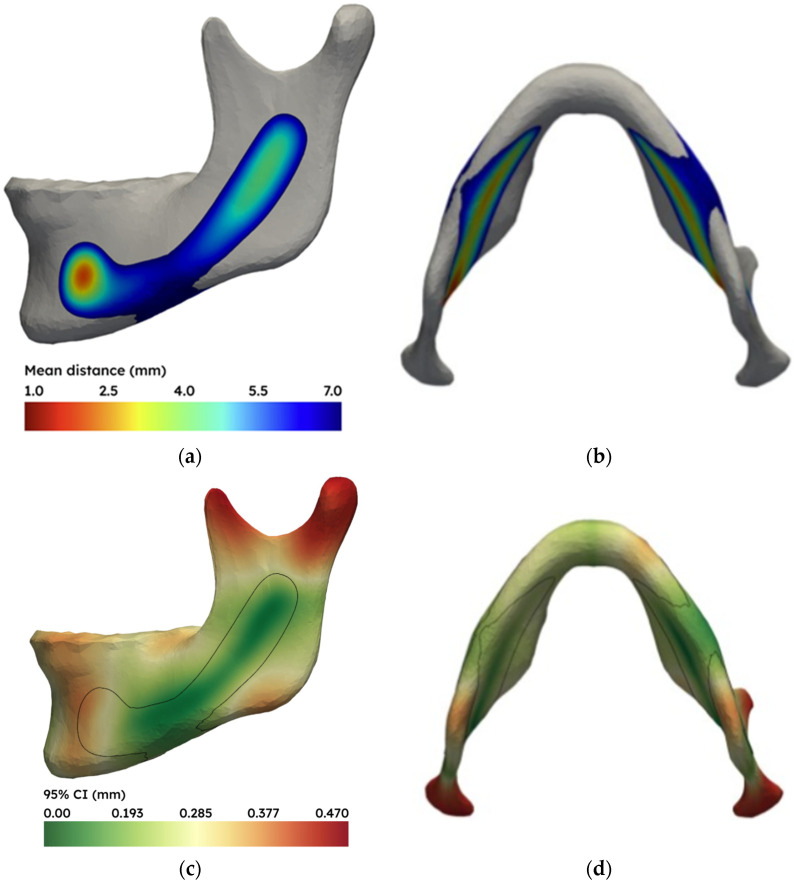
(**a**) Colourmap of the average distance from the cortex of the 428 mandibles to the mandibular nerve canal plotted on the mean mandible shape, and (**b**) the same image seen from a caudal view. Red corresponds to a small distance (1.0 mm), blue to a larger distance (7.0 mm), and grey to distances larger > 7 mm. (**c**) Visualisation of the half-width of the 95% confidence interval on the mean nerve-to-cortex distance, reflecting the statistical precision of the population average at each surface point. Green is very precise (0.0 mm), red is less precise (0.47 mm). The black contour line shows the iso-line of the 7 mm mean nerve to cortex distance. This iso-line corresponds with the contour of the (**a**,**b**) distance colourmap. (**d**) Caudal view of the same analysis.

The shortest distance from each surface point of the cortex to the mandibular nerve canal was calculated for all 428 mandible models. Each cortical point of the resulting list of 428 distance measurements was averaged, and the result was plotted as a colourmap on the mean mandible model (see Figure 3a,b). Figure 3c,d show the half-width of the 95% confidence interval of the mean at each surface point.

### 3.3. Statistical Analysis

**Figure 4:**
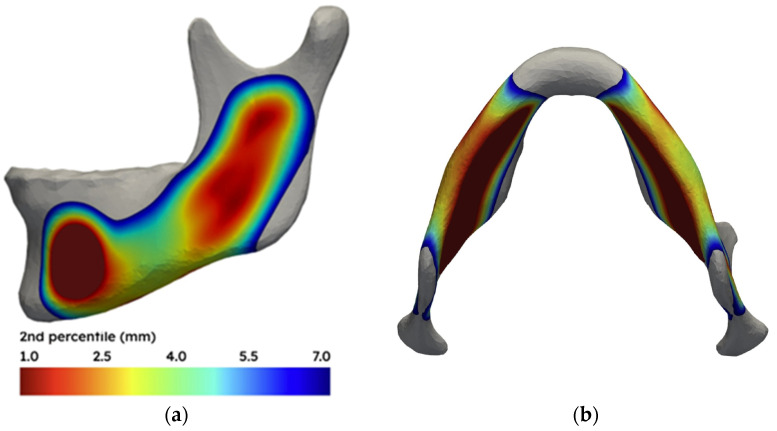
(**a**) Visualisation of the 2nd percentile distance from the mandibular cortex to the mandibular nerve canal. At each location, at least 98% of patients have a greater nerve-to-cortex clearance than the value shown. Red indicates a small clearance (<1 mm), and blue indicates a large clearance (up to 7 mm). Values below 1 mm (e.g., in the region of the mental foramen) are shown in red, and distances > 7 mm are in grey. (**b**) Caudal view of the same analysis.

The deviation in each cortex-to-nerve distance point’s measurement was tested for normal distribution using the Shapiro–Wilk test (*p* = 0.05). *p*-values were adjusted with the Benjamini–Hochberg false-discovery-rate (FDR) correction (α = 0.05). After correction, a substantial proportion (70%) of surface points still showed significant deviation from normality. To visualise the ‘worst plausible distance’ from the cortex to the nerve, we used the 2nd percentile of the distance distribution at each vertex. This value represents the minimum clearance that at least 98% of individuals exceed at a given location. Figure 4a,b shows these 2nd percentile distances as a colour-coded map on the mean mandible shape, highlighting areas where the nerve consistently lies deeper relative to the cortex. This non-parametric approach avoids assumptions about the normality of the distribution, which is supported by Shapiro–Wilk testing (*p* ≈ 0.05).

### 3.4. Osteotomy-Specific Distance Measurements

Table 1 presents the buccal and inferior clearance measurements for each of the four BSSO osteotomy designs shown in Figure 1.

The pre-notch osteotomy, corresponding to the second molar region, demonstrated the largest buccal clearance, with a mean distance of 7.6 mm (95% CI: 7.4–7.7 mm) and a 2nd percentile of 4.1 mm. In contrast, the post-gonial osteotomy showed the smallest buccal clearance, with a mean of 5.7 mm (95% CI: 5.6–5.9 mm) and a 2nd percentile of only 1.6 mm, indicating that in 2% of patients the buccal bone covering the nerve at this location is less than 1.6 mm.

Regarding inferior clearance, the post-gonial and post-notch osteotomies demonstrated substantially greater distances from the nerve to the inferior border compared with the premolar and pre-notch regions. The post-gonial osteotomy had a mean inferior distance of 16.6 mm (95% CI: 16.4–16.8 mm) with a 2nd percentile of 10.9 mm, whereas the pre-notch osteotomy had a mean inferior distance of 6.8 mm (95% CI: 6.6–6.9 mm) with a 2nd percentile of 3.5 mm.

## 4. Discussion

This study presents a comprehensive three-dimensional analysis of the inferior alveolar nerve (IAN) course in 428 consecutive digitally planned BSSO cases. The results confirm substantial variability in nerve position. We found that in 98% of patients, the mandibular nerve canal lies approximately 5 mm from the buccal cortex in the second molar region, thereby identifying this as a consistently safe zone for a buccal osteotomy. In contrast, the mandibular nerve canal in the vertical ramus and premolar areas often runs within less than 2.5 mm of the cortical bone.

Our findings are in line with an earlier cadaveric study by Promma et al., who recommended limiting sagittal cuts to approximately 4.5 mm in the ramus region, 6.5 mm at the second/third molar, and 5 mm at the first molar [6]. Our large-scale CBCT-based data confirms these cadaveric values and extends them into a continuous three-dimensional risk map. Moreover, most studies using CBCT scans measured the buccolingual distance on the cross-sectional view of the CBCT, whereas our method uses true 3D measurements.

Previous studies have emphasised the complex, ‘wavy’ course of the mandibular nerve canal through the mandible [5,6,7,8,9,11]. Our data corroborate this concept and add a continuous map of risk gradients, showing that buccal bone clearance is greatest in the second molar region and diminishes toward the premolar region and posterior ramus. Although our population-based ‘heat map’ delineates relatively safer versus riskier areas, it cannot replace patient-specific planning. Furthermore, factors such as cortical bone thickness and intraoperative deviations from the planned osteotomy trajectory can influence the actual risk of direct nerve injury, underscoring the importance of preoperative identification of the safest osteotomy region through 3D analysis.

The second molar region offers a relatively large margin to the mandibular nerve canal in most patients. This region is therefore generally preferred to minimise the risk of direct nerve injury from surgical instrumentation. However, there are valid anatomical or biomechanical reasons to consider a more posteriorly directed osteotomy [12,13]. Verweij et al. introduced an angled osteotomy technique with an oblique buccal cut aimed at the masseteric tuberosity [12]. Their cadaveric study showed that this design led to more posterior lingual fractures and fewer bad splits. The oblique osteotomy also resulted in less frequent entrapment of the IAN in the proximal segment. Although these trends did not attain statistical significance, they support the potential advantages of this design. Our data show that the nerve often lies closer to the outer surface of the buccal cortex of the vertical ramus (Figure 4), which increases the risk of direct nerve injury, especially when using traditional instruments like drills or saws. Wolford also highlighted this concern with the Verweij approach [14]. While the angled cut may reduce fracture torque and improve split predictability, cautious patient-specific assessment is important due to the reduced buccal nerve clearance observed in this study. Valls-Ontañón et al. reviewed the conventional BSSO pre-notch, oblique post-notch, and horizontal post-gonial buccal osteotomy techniques [5]. Valls-Ontañón et al. reported a lower risk of nerve injury when using a more posterior post-notch or post-gonial buccal osteotomy. However, we found that the IAN in the vertical ramus runs closer to the buccal cortex but at a greater distance from the inferior border than in the second molar region.

Interestingly, our measurements suggest that in the mandibular angle regions, the distance from the nerve to the inferior border tends to be larger than in the second molar region. A posteriorly directed osteotomy may therefore allow earlier and more direct visualisation of the mandibular canal during surgery, potentially offering better control over nerve exposure compared with more anterior cuts, where the nerve may remain concealed until later in the procedure. This observation highlights that, when carefully planned, posterior osteotomies may present both challenges and opportunities for improved intraoperative nerve management.

The osteotomy-specific measurements presented in Table 1 quantify these anatomical patterns. The pre-notch osteotomy in the second molar region demonstrated the most favourable buccal clearance, with a mean of 7.6 mm. In contrast, the post-gonial osteotomy showed a mean buccal clearance of only 5.7 mm, reflecting the limited margin available in this region. This relationship is reversed for inferior clearance: the post-gonial trajectory offers a mean inferior clearance of 16.6 mm, compared with only 6.8 mm at the pre-notch site. These data indicate an inverse relationship between buccal and inferior clearance along the mandible, with practical implications for surgical planning. Surgeons opting for a posteriorly directed osteotomy to reduce inferior border notching or facilitate the correction of complex vertical asymmetry should be aware of the reduced buccal safety margin and may benefit from early visualisation of the nerve through the inferior approach. Conversely, osteotomies in the pre-notch region offer greater buccal clearance but require careful attention to the inferior border, where the nerve lies closer to the cortical surface.

Methodologically, this study’s strength lies in the use of a large clinical CBCT dataset from patients who underwent BSSO surgery. This approach has advantages over cadaveric studies, in which nerve morphology may be affected by postmortem changes or advanced donor age. By tracing the mandibular nerve canal of osteotomy patients in three dimensions, and presenting a 3D analysis of the results, we achieved a comprehensive representation of the mandibular nerve anatomy that is relevant to BSSOs.

Nevertheless, our work is subject to limitations inherent in CBCT imaging. As Agbaje et al. showed, CBCT tends to under-segment the nerve compared with an MRI, which may lead to a slight underestimation of true canal dimensions [11]. In this study, we used a standardised 2.5 mm diameter for tracing the IAN canal, which is in line with the median nerve diameter found in healthy individuals [10]. While canal diameter varies among patients, accurately tracing the three-dimensional boundaries of the canal, let alone the nerve itself, on CBCT remains challenging. The standardised diameter was therefore chosen to enable consistent population-level analysis. Consequently, the exact safety margins presented in this study should be interpreted with caution, and thorough patient-specific preoperative planning with individual IAN canal mapping remains essential. This study focused on the distance of the IAN canal to the outer surface of the bone and did not incorporate cortical bone thickness. A future study incorporating cortical bone thickness into the safety margin calculation would provide a more complete assessment of the bone available for safe osteotomy instrumentation. Additionally, this study did not stratify the data by demographic or clinical subgroups such as sex, age, or skeletal class (Class II versus Class III). It is possible that nerve position and safety margins differ between these groups, and future studies should investigate whether population-specific risk maps could further refine preoperative planning.

Furthermore, we excluded 12 patients as we could not trace one of the nerves. However, especially for those cases where the nerve is not traceable but a BSSO needs to be performed, the current results could be used as a guideline on where to perform the osteotomy. Postoperative neurosensory outcomes were not available for this retrospective cohort, and correlating anatomical findings with clinical nerve injury would require a prospective study design that distinguishes direct intraoperative trauma from indirect injury mechanisms. Given the observed anatomical variability and the known risk of nerve injury during a BSSO, our findings suggest that routine preoperative CBCT imaging combined with 3D segmentation of the mandibular nerve canal may benefit BSSO planning, especially when directing the osteotomy towards the gonial angle. However, this study demonstrates anatomical patterns rather than clinical outcomes, and prospective studies are needed to confirm their clinical utility.

## 5. Conclusions

This study presents the first population-based three-dimensional map of inferior alveolar nerve clearance in BSSO patients, revealing significant anatomical variability across the mandible. The pre-notch osteotomy in the second molar region offers the greatest buccal bone clearance, whereas posteriorly directed osteotomies provide larger inferior margins but reduced buccal safety clearance. These data may serve as a reference when patient-specific nerve tracing is not feasible and could guide the safe adoption of evolving osteotomy techniques. Future prospective studies are warranted to determine whether integration of these anatomical findings into surgical planning reduces postoperative nerve injury.

## Figures and Tables

**Table 1:** Buccal and inferior distance measurements (in mm) from the mandibular cortex to the inferior alveolar nerve canal for four BSSO osteotomy designs. Values represent the mean, 95% confidence interval (CI), and 2nd percentile (P2) across 428 patients.

Osteotomy Design	Buccal Distance (mm)			Inferior Distance (mm)		
	Mean	95% CI	P2	Mean	95% CI	P2
Anterior	6.9	[6.7–7.1]	3.5	7.4	[7.3–7.6]	4.1
Pre-notch	7.6	[7.4–7.7]	4.1	6.8	[6.6–6.9]	3.5
Post-notch	6.4	[6.3–6.6]	3.0	15.5	[15.2–15.7]	8.4
Post-gonial	5.7	[5.6–5.9]	1.6	16.6	[16.4–16.8]	10.9

## Data Availability

Data are available on reasonable request to the corresponding author.
